# The embedded tumour: host physiology is important for the evaluation of tumour growth

**DOI:** 10.1038/sj.bjc.6601394

**Published:** 2003-12-09

**Authors:** I M M van Leeuwen, C Zonneveld, S A L M Kooijman

**Affiliations:** 1 Department of Theoretical Biology, Faculty of Earth & Life Sciences, Vrije Universiteit, De Boelelaan 1085, 1081 HV Amsterdam, The Netherlands

**Keywords:** tumour growth rate, cancer cachexia, DEB theory, energy expenditure, calorie restriction, tumour doubling time

## Abstract

The growth potential of a tumour can significantly depend on host features such as age, cell proliferation rates and caloric intake. Although this is widely known, existing mathematical models for tumour growth do not account for it. We therefore developed a new model for tumour growth, starting from a mathematical framework that describes the host's physiology. The resulting tumour-in-host model allowed us to study the implications of various specific interactions between the energetics of tumour and host. The model accounts for the influence of both age and feeding regimen of the host organism on the behaviour of a tumour. Concerning the effects of a tumour on its host, it explains why tumour-mediated body-weight loss is often more dramatic than expected from the energy demands of the tumour. We also show how the model can be applied to study enhanced body-weight loss in presence of cachectic factors. Our tumour-in-host model thus appears a proper tool to unite a wide range of phenomena in tumour–host interactions.

Mathematical models for tumour growth have been widely used in different subdisciplines, such as cancer risk assessment (Dewanji *et al*, 1991; Sherman and Portier, 2000), cancer biology (Laird, 1964; Ward and King, 1999), cancer treatment (Thomlison and Gray, 1955; Adam and Bellomo, 1997), and oncological decision making (Friberg and Mattson, 1997). Since the first models for tumour growth were published (Mayneord, 1932; Winsor, 1932; Von Bertalanffy, 1957), they have become more detailed and, consequently, more complex (Groebe and Mueller-Klieser, 1991; Ward and King, 1997). Most classic and modern approaches share at least one feature, though: both describe the increase in size of an independent ‘entity.’ The models are therefore adequate to analyse, for instance, data on tumour spheroids growing *in vitro*. Their use to describe data on tumours growing *in vivo* may be less warranted because of interactions between tumour and host. The aim of this article is to develop a mathematical model to explore such interactions between the growth of a tumour and the physiology of the host organism.

We based our model on well-recognised interactions between tumour growth, energy homeostasis, utilisation of stored energy by tumour and host and cancer cachexia. The formulation in terms of a mathematical model has several benefits. First, it forces us to specify quantitative formulations about the interactions, which improves testability of the hypotheses involved. Second, because the model asks for an overall view of a number of processes and their inter-relationships, it can offer insights that complement those arising from individual experimental studies. Finally, model simulations allow to switch on or off particular hypothetical mechanisms easily, so that we can evaluate their impact on and relevance for the expected outcome.

The article is organised as follows. First, we introduce the dynamic energy budget (DEB) theory (Kooijman, 2000,^2001^), which provides quantitative expressions for fundamental physiological features and processes, such as food consumption, body growth, metabolic rate, and ageing. We then extend this theory to account for tumour growth. Second, with the aid of computer simulations, we show that tumour growth can significantly depend on host physiology and *vice versa*. Regarding the influence of the host on tumour behaviour, we focus on the implications for the tumour of differences in host energetics associated with host age and host caloric intake. Thereafter, we study the decrease in body weight associated with the increase in size of a tumour. Finally, we discuss several implications of the results obtained. The Appendix contains additional information on the mathematical formulation of the model as well as on the fitting procedures and parameter values.

## MATERIALS AND METHODS

### Introduction to the DEB theory

To model the interaction between tumour and host, we need a general framework describing the physiology of the host organism. Such a framework is provided by the DEB theory. The theory starts with a set of rules to characterise an individual organism, based on fundamental mechanisms that all organisms seem to have in common. From these rules, the theory derives quantitative expressions for sundry physiological processes. In this article, we explain only those aspects of the theory indispensable to understand our model for tumour growth. A more complete, though still qualitative, introduction to the theory can be found in Kooijman (2001), while Kooijman (2000) provides an exhaustive formulation.

Figure 1

**Figure 1:**
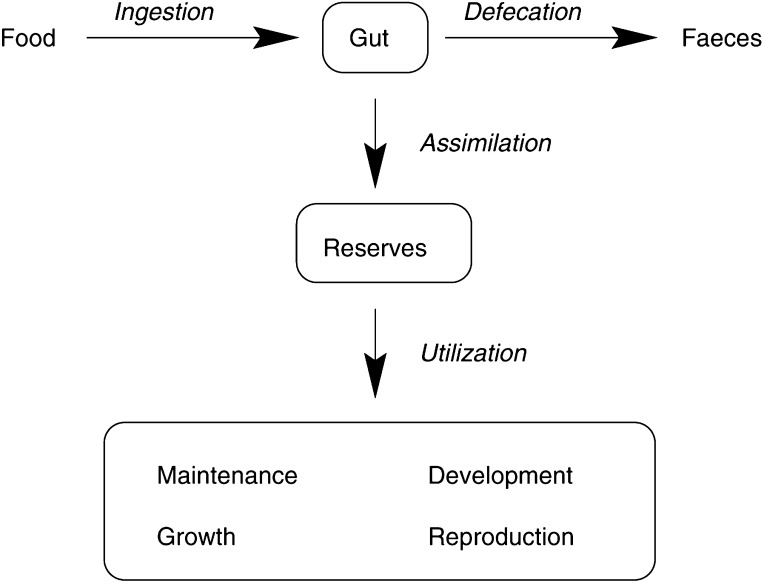
Energy fluxes in an individual organism, according to the DEB theory. Food is conceived as material that bears energy. Part of this energy is taken up via blood and delivered to the reserves. Energy required to carry out the various physiological processes is obtained from these reserves.

shows the basic outline of the DEB framework. According to this framework, the body consists of two components, namely structural biomass and reserve compounds. The latter pool comprises compounds characterised by a high mobility. The reserve dynamics follows from the supply and demand of the available resources. Structural biomass can be conceived as volume, hence it is denoted by *V*(*t*). Both body components have, by assumption, a constant, but not necessarily identical, chemical composition. As the relative amount of reserves and structure can vary, the composition of the total body can vary. For instance, during fasting the body loses predominantly reserves, so that the overall composition of the body changes.

### The *κ*-rule

Maintenance costs play a key role in our model. Maintenance comprises a range of different processes, among which are protein turnover, heating, maintenance of membrane concentration gradients, and muscle tension levels. The costs of such processes should be distinguished from the costs of growth, development, and reproduction, as was already concluded in Duclaux (1898). Since then, the importance of maintenance processes has become widely accepted (Pirt, 1965; Payne and Waterlow, 1971; Canolty and Koong, 1976). The DEB theory assumes that maintenance costs per unit structural volume per time unit, [*M*], are constant, which implies that total maintenance costs amount to *M*(*t*)=[*M*]*V*(*t*) per time unit. This assumption leads to a relationship between body size and respiration that accounts for both growth and maintenance. This prediction is well supported by experimental data concerning the scaling of respiration with body size (Kooijman, 2000).

The DEB theory assumes that somatic processes (growth and maintenance) and reproductive processes (development and reproduction) take place in parallel. This is supported by the observation that some species start reproduction while they are still growing; others start reproduction well after reaching adult size. Yet in both species, growth levels off in the same way. This implies that the onset of reproduction cannot be the cause of the cessation of growth.

According to the so-called *κ*-rule, an individual spends a fixed fraction *κ* of the available energy on somatic processes (growth and maintenance), whereas it spends the remainder fraction on reproductive processes (development, maintaining the degree of differentiation, and reproduction). The part of the *κ*-rule concerning growth can be written as


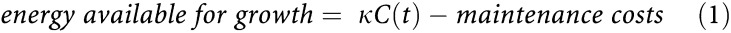


with *C*(*t*) being the utilisation rate at time *t*; the utilisation rate is the rate at which energy is mobilised from the reserves and is made available for physiological processes (see Figure 1). All the quantities in equation (1) are expressed per time unit. Thus, equation (1) is an energy *rate* balance, rather than an energy balance. This applies to all similar equations in our article.

To stay alive, an animal has to give maintenance priority over growth. Increase in size consequently ceases when all energy available for maintenance and growth is spent on maintenance only. Maintenance thus determines the ultimate size an organism can reach. The costs of growth are the same for each unit increase in size. Thus, costs of growth per time unit are proportional to the increase in structural volume: *G*(*t*)=[*G*]d*V*/d*t*, with [*G*] being a constant. With the energy available for growth equation (1)), the organism's size thus changes according to


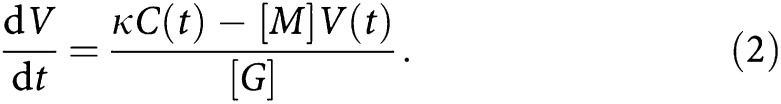


The DEB model provides a quantitative expression for the utilisation rate *C*(*t*) (see Appendix A). When food availability remains constant and food intake is proportional to a body surface area, equation (2) reduces to the well-known Von Bertalanffy growth equation (Von Bertalanffy, 1957). This equation fits growth curves of a wide variety of animal species that do not change in shape during growth (Kooijman, 1988).

### Generalised *κ*-rule

In the introduction to the *κ*-rule above, we treated the animal's structure as a single variable. Since we want to describe tumour growth within the DEB framework, we have to expand the basic formulation. Suppose we zoom in on a cell that changes into a tumour cell. From an energetic point of view several things may happen. First, because tumour tissue is generally less differentiated than other tissues, tumour growth and maintenance costs per tumour volume may be lower, allowing tumour cells to proliferate faster than normal cells. However, because a tumour is a part of the body that has run out of control, a second energetic aspect may also change: a tumour cell may consume more than its share of the available energy, at the expense of other tissues. In other words, tumour cells may become gluttonous, taking what they want, and leaving the left-over available to the body proper. Thus, in our approach to tumour growth, mutations can lead to hyperplasia by decreasing the costs of somatic processes (maintenance or growth) or by increasing the energy supply per cell.

To model tumour growth dynamics, we need some additional variables and parameters. In addition to body size *V*, we consider tumour size *V*_*u*_. Obviously, to survive and proliferate, the tumour has to obtain nutrients from the host. We characterise the gluttony of the tumour by a coefficient *μ*_*u*_. If *μ*_*u*_=1, each tumour cells demands the same amount of energy as an average normal cell; if *μ*_*u*_>1, then a tumour cell takes more than an average body cell. Below, we will argue that the gluttony coefficient *μ*_*u*_ plays an important role in determining the aggressiveness of a tumour.

The growth rate of a tumour is not only determined by the ability of the tumour to exploit the host's resources, but also by the tumour's maintenance and growth investments. We assume that the tumour appropriates a fraction *κ*_*u*_(*t*) of the energy that the host has available for somatic processes. This assumption implies that tumours have priority for the resources over the host, which is supported by experimental evidence (Cameron *et al*, 1979). The *κ*-rule above equation (1)) can now be extended to account for the energetics of the developing tumour:


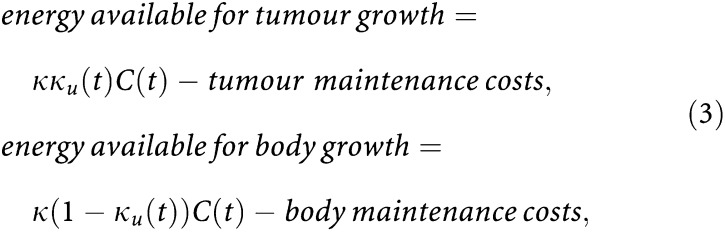


where *C* is the rate of energy mobilisation from the reserves. Like in equation (1), all quantities are expressed per time unit. Experimental observations support that the tumour's energy demand increases with tumour size. This means that *κ*_*u*_ is a function of tumour size. We assume that


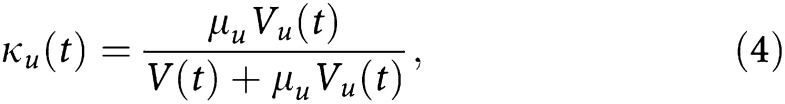


so that *κ*_*u*_, like *κ*, takes values between 0 and 1. Our assumption implies that at small tumour size, the fraction of the resources appropriated by the tumour is approximately proportional to tumour size. As the tumour becomes larger, the fraction still increases, but at a diminishing pace. The energy-allocation rules above (equations (3) and (4)), together with the expressions for the tumour's maintenance and growth costs, completely specify the growth of a tumour. Appendix A outlines further details on the model equations.

## RESULTS

In this section, we analyse the implications of our approach with the aid of computer simulations. For this purpose, we first need to have a set of values for the physiological parameters. These values differ between species, so we had to choose a particular species. As our target species we chose the rat, because many data relevant to our study pertain to rodents. Moreover, since the rat is a typical model species in cancer research, this choice may facilitate testing of our predictions.

**Figure 2:**
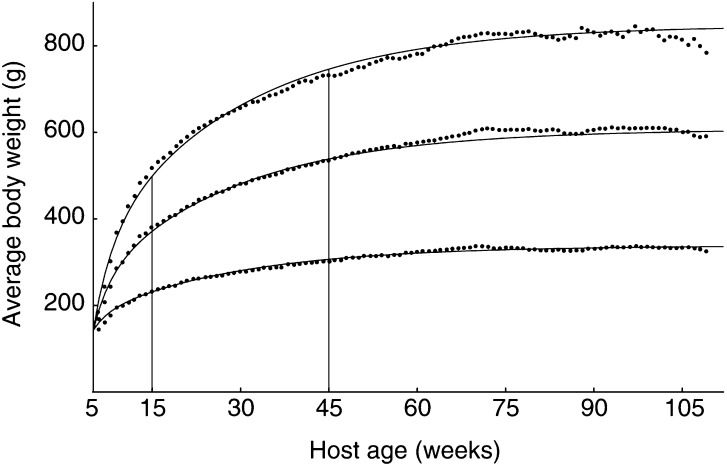
Growth of Sprague–Dawley male rats. From top downwards: food available *ad libitum*, 25% caloric restriction, and 55% caloric restriction. Dots represent data from Hubert *et al* (2000). The animals were 5 weeks old at study initiation. We fitted the three data sets simultaneously, varying only food supply among the diet groups. For information on the fitting procedure and the five estimated parameter values, see Appendix. Tumorigenesis may occur, for instance, at age *t*_*i*1_=15 or at age *t*_*i*2_=45 weeks. The vertical lines indicate these moments.

As explained in Van Leeuwen *et al* (2002), for tumour-free laboratory rodents after weaning it is warranted to assume constant food consumption. In our approach, this experimental observation replaces the DEB-based assumption that food uptake increases with body size. To obtain the required host parameter values, we fitted the resulting model to data on male rat body growth from a study by Hubert *et al* (2000). This study includes three groups of 60 male rats exposed to *ad libitum* feeding, 25% caloric restriction, and 55% caloric restriction. Figure 2

shows the growth curves corresponding to the estimated parameter values. Information on the fitting procedure can be found in Appendix B.

**Figure 3:**
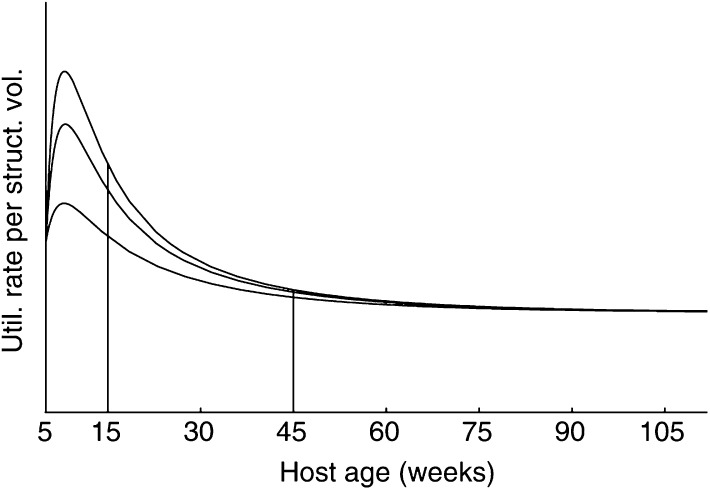
Model simulation of the energy-expenditure rate per structural volume, [*C*]=*C*/*V*. From top downwards: food available *ad libitum*, 25% caloric restriction, and 55% caloric restriction. Tumorigenesis may occur, for instance, at age *t*_*i*1_=15 or at age *t*_*i*2_=45 weeks. The vertical lines indicate these moments.

Once values for the parameters characterising the organism are known, we are able to predict the behaviour of the utilisation rate as a function of age. In this article, we will show that the utilisation rate per structural volume ([*C*]=*C*/*V*), rather than the utilisation rate itself, is important for tumour growth. As can be seen from Figure 3

, caloric intake significantly affects [*C*] at the beginning. After some time, however, the body adapts to low food availability and the difference in [*C*] with food availability disappears. This is in agreement with the experimental observation that differences in energy expenditure per lean body mass disappear with long-term caloric restriction (Ramsey *et al*, 2000).

### Tumour growth

In addition to the choice of the rat physiological parameters values, we also need to characterise the tumour by choosing appropriate parameter values. Because of the lack of adequate tumour growth data, we choose these values with an eye on host parameter values. Basically, three parameters characterise the tumour: its coefficient of gluttony *μ*_*u*_, its growth costs [*G*_*u*_], and its maintenance costs [*M*_*u*_]. It is the values of these parameters that determine the ability of a tumour to outgrow host tissues. Tumour cells, for instance, may be more successful extracting nutrients from the blood than normal cells (i.e., *μ*_*u*_>1). Moreover, because tumour cells have no fine-tuned morphology, it seems likely that tumour growth costs are less than host growth costs (i.e., [*G*_*u*_]<[*G*]). The same logic applies to tumour maintenance costs (i.e., [*M*_*u*_]<[*M*]).

**Figure 4:**
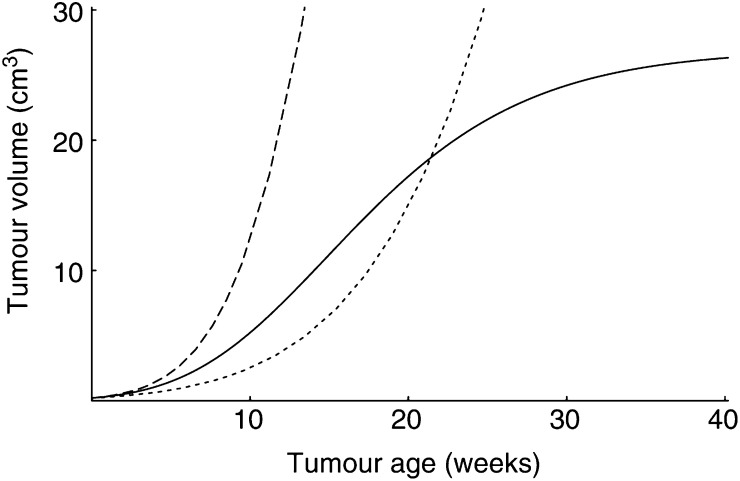
The shape of the tumour growth curve depends on the relative values of the tumour and host parameters. For any curve: [*G*_*u*_]<[*G*]. Solid line: *μ*_*u*_>1 and [*M*_*u*_]=*μ*_*u*_[*M*]; dotted line: *μ*_*u*_=1 and [*M*_*u*_]<[*M*]; broken line: *μ*_*u*_>1 and [*M*_*u*_]=[*M*]. Tumorigenesis at age *t*_*i*1_=15 weeks in an *ad libitum* fed host (see Figure 2). For further information on the parameter values, see Appendix B.

To obtain the expressions above (equations (3) and (4)), we did not make *a priori* assumptions on the shape of the tumour growth curve. Our simulations show that both saturating and nonsaturating growth patterns are possible (see Figure 4

). The relevant quantities that determine the growth pattern are the maintenance costs of tumour cells compared to that of host cells and the coefficient of gluttony. Hence, it turns out that a tumour can only grow if [*M*_*u*_] is smaller or equal to *μ*_*u*_[*M*]. Moreover, only if [*M*_*u*_]=*μ*_*u*_[*M*] holds, it has an S-shaped growth curve. In contrast, if [*M*_*u*_]>*μ*_*u*_[*M*], the tumour dies off.

### Influence of host on tumour

#### Effect of host age on tumour growth

Cancer incidence rates clearly vary with age. Yet, the influence of host age is not restricted to the tumorigenesis phase. Several studies indicate that tumour growth rates also depend on host age. For example, Peer *et al* (1993) found that breast cancers grow slower in old than in young human females. Pili *et al* (1994) inoculated Engleberth–Holm–Swarm (EHS) carcinoma cells into mice of different ages. They reported that EHS tumours develop faster in young than in old mice (Pili *et al*, 1994). Moreover, rapid tumour growth resumed upon transfer of tumour tissue from old animals into young animals. Likewise, Donin *et al* (1997) found a decreased growth potential of B16 melanomas in middle-aged *vs* young mice. Besides reduced growth rates, a less aggressive behaviour of tumours has been reported in old as compared to young hosts (Holmes, 1989).

**Figure 5:**
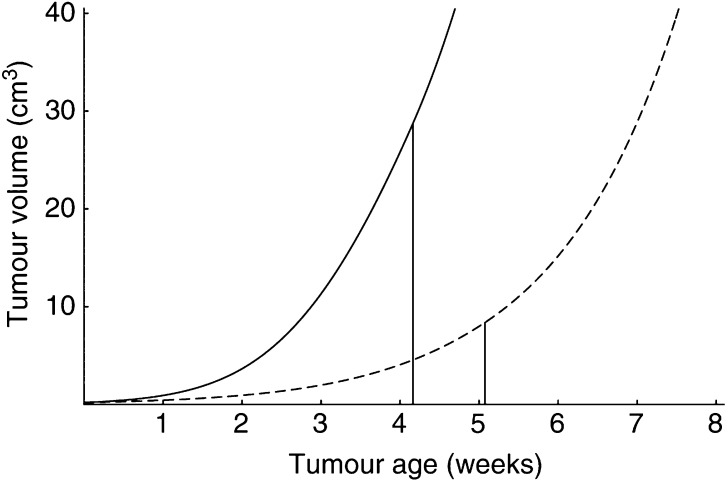
Tumour growth is influenced by changes in energetics during the host's lifespan. Solid line: growth of a tumour early in the host's life (transplantation at age *t*_*i*1_=15 weeks); broken line: growth of the same tumour later in life (transplantation at age *t*_*i*2_=45 weeks). Tumour parameters values: *μ*_*u*_>1, [*M*_*u*_]<[*M*], and [*G*_*u*_]<[*G*]. Whereas Figure 4 depicts the change in size of three slowly growing tumours, this figure corresponds to a more aggressive tumour. The interpretation of the vertical lines will be clarified later on. For further information on the parameter values, see Appendix B.

To study the effect of host age on tumour progression with our modelling approach, we considered two *ad libitum* fed male rats of ages 15 and 45 weeks, respectively (see Figure 2). We simulated the implantation of a tumour cell clone (*V*_*ui*_=0.2 cm^3^; 10 million cells approximately) of the same type of tumour in both animals. The resulting tumour growth patterns are shown in Figure 5

. The behaviour of the tumours differs significantly. As we did not incorporate in our model any phenomena related to the ageing process *per se*, the predicted age-related differences in tumour growth can be attributed to changes in the energetic state of the host during its lifespan. Figure 3 shows that the host energy expenditure per structural volume diminishes with age. This results in a lower energy availability for the tumour in old *vs* young host, leading to slower tumour growth in the older hosts.

#### Effect of caloric restriction on tumour growth

Another aspect of tumour–host interactions is the effect of host nutrition on tumour growth. In the context of the DEB theory, physiological processes such as energy expenditure, body growth, and ageing depend on food intake (Van Leeuwen *et al*, 2002). As we developed our model for tumour growth within this framework, our approach naturally accounts for food consumption. The model is thus suited to study quantitatively, for example, the influence of host caloric intake on the behaviour of a tumour.

Figure 6

**Figure 6:**
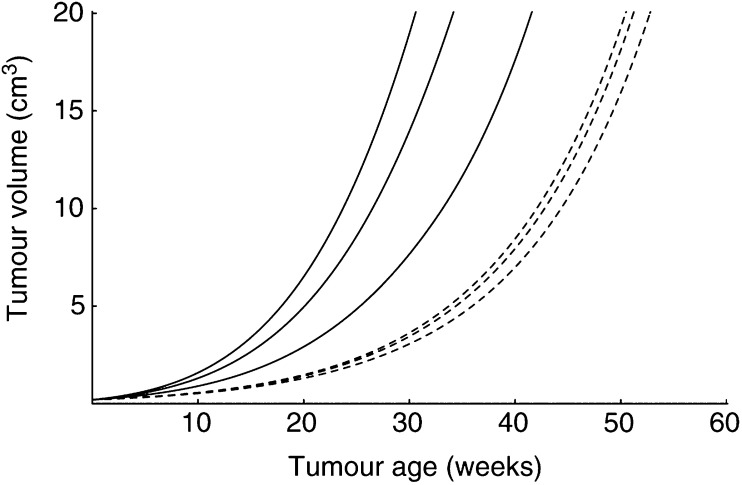
Food consumption affects tumour growth. Solid lines: tumour implantation after a short exposure to caloric restriction (*t*_*i*1_=15 weeks); dotted lines: implantation of the same tumours after long time exposure to the same levels of caloric restriction (*t*_*i*2_=45 weeks). Same tumours as in Figure 5. For each set of three curves, from left to right, food available *ad libitum*, 25% caloric restriction, and 55% caloric restriction.

shows the growth of a tumour in hosts exposed to the same levels of caloric restriction that underly the different growth curves depicted in Figure 2. The solid lines represent the growth of the tumour in three hosts that have been exposed to the feeding regimen for only 10 weeks; the broken lines correspond to tumorigenesis after 40 weeks exposure. There are thus two variables in this simulation. First, age at tumour transplantation, and second, duration of the exposure to caloric restriction before tumour transplantation. The effect of age was already discussed in Figure 5. Figure 6 adds to this the impact of the different levels of caloric restriction. Based on differences in the disparity of the three curves for each age of transplantation, we conclude that short-term caloric restriction has far greater influence on tumour development than long-term caloric restriction.

As explained above, in our model the growth capacity of a tumour depends on the host's rate of energy expenditure per structural volume, [*C*]. Figure 3 shows that food restriction results in a diminished [*C*]. We therefore predict that a tumour grows slower in calorically restricted animals than in *ad libitum* fed ones. However, as can also be seen from Figure 3, the body adapts to low food availability and the differences in [*C*] become smaller after exposure to long-term caloric restriction. Consequently, the effect of caloric restriction on tumour growth fades away during long-term caloric restriction. For this reason, the broken lines in Figure 6 are closer to each other than the solid lines.

### Influence of tumour on host

#### Effect of tumour growth on body weight

We now pay attention to the implications of tumour growth for host physiology. As the tumour exploits the resources of the host organism, the latter disposes of less energy to carry out normal physiological processes. As maintenance always has priority over growth, the energy spending-cut initially results in a decrease of the host growth rate. If it decreases to zero and tumour size still increases, the host has two ways to survive while satisfying the tumour's energy demand: (a) reduce its own maintenance investment and (b) degrade structural biomass. The former entails that not all required maintenance processes are carried out, which may lead to serious physiological problems and predispose for disease. The latter results in loss of, for instance, skeletal muscle, which may ultimately lead to death.

Although the generalised *κ*-rule (equations (3)) allows for body-weight loss, there are two reasons why it would be inappropriate to use these equations to describe tissue degradation. First, if these equations were used, all energy originally invested in ‘building’ a unit biomass would be regained, which is thermodynamically impossible. Second, equations (3) imply that the host reutilises all energy released from tissue degradation to pay its own maintenance costs. This contradicts accepted knowledge, indicating that both host and tumour benefit from the released resources. We therefore have to account explicitly for tumour-mediated body-weight loss.

**Figure 7:**
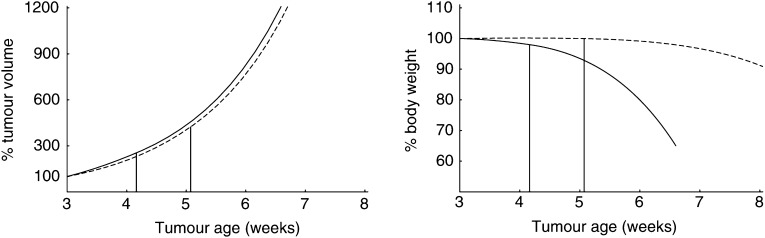
Tumour growth affects host body weight. The results concern the same computer-simulation study as Figure 5. Left: tumour size as a fraction (in %) of its volume 3 days after tumour implantation. Right: body weight as a fraction (in %) of the host's body weight 3 days after tumour implantation. The vertical lines indicate when tumour-mediated loss of structural biomass starts. The earlier decrease in total body weight (see right panel) is due to a depletion of reserve materials. Tumour transplantation took place at age *t*_*i*_=15 weeks (solid lines) and *t*_*i*_=45 weeks (broken lines) in *ad libitum* fed hosts.

The generalised *κ*-rule (equation (3)) can easily be extended to account for the loss of body weight often observed in tumour-bearing organisms. Above we argued that a tumour has priority over the available resources. This implies that it also demands a fraction *κ*_*u*_ of the energy obtained from the loss of structural biomass. The host reutilises the remainder to pay its own maintenance costs. When no energy is available for body growth, equations (3) can be written as


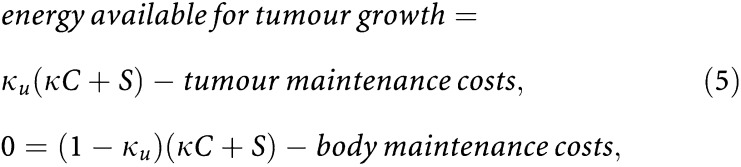


where *S* represents the rate at which energy is regained from the degradation of structural biomass. We assume that *S*(*t*)=−*ω*[*G*]d*V*/d*t*, which means that the amount of energy that becomes available per time unit is proportional to the tissue degradation rate (notice that, because the host loses structural volume, d*V*/d*t* is negative and, consequently, *S* is positive). The parameter *ω* is an efficiency coefficient. The thermodynamic upper limit *ω*=1 means 100% efficiency, which, however, can never be achieved. In the realistic case that *ω*<1, part of the degraded structural biomass is actually wasted. Figure 7

shows the predicted body-weight loss associated with the growth of the tumours depicted in Figure 5. According to our model, tumour-mediated decrease in body weight involves a depletion in both structure and reserve materials. This is in agreement with the observation that most cancer patients suffer a progressive decrease in both adipose tissue and skeletal muscle.

Cancer patients with the same tumour type can significantly vary in the extent to which they suffer from body-weight loss. Such variations also occur in the context of our model. For instance, Figures 5 and 7 show the development of the same tumour in two hosts that differ in age and, consequently, also in size and energetic state. The time at which the loss of structural biomass begins, *t*_*s*_, is indicated with a vertical line. Notice that total body weight (Figure 7, right panel) begins to decrease before *t*_*s*_, which is due to an earlier depletion of reserve materials. As can be seen from Figure 5, in the young host, loss of structural biomass initiates when the tumour reaches a size of 28.7 cm^3^. In contrast, in the older host, it starts when the tumour has a size of only 8.4 cm^3^. The time delay between tumour implantation and manifestation of structural-biomass loss also varies with host age. Indeed, in the young it concerns a delay of 4.2 weeks, whereas in the older host it concerns a delay of 5.1 weeks. We conclude that body-weight loss is determined by both host and tumour, rather than by the tumour alone.

### Cachexia

The loss of body weight shown in Figure 7 is due to interactions between the energetics of tumour and host. A tumour may enhance body-weight loss by producing (or inducing the production of) factors that interact with the host. This may lead to the syndrome known as cancer cachexia, which is a common cause of morbidity and mortality in cancer patients. Among the proposed cachectic factors are several cytokines (Matthys and Billiau, 1997), a lipid-mobilising factor (Tisdale, 2000), and a proteolysis-inducing factor (Tisdale, 2001). The degradation of structural biomass induced by such factors can be incorporated into the generalised *κ*-rule as follows:


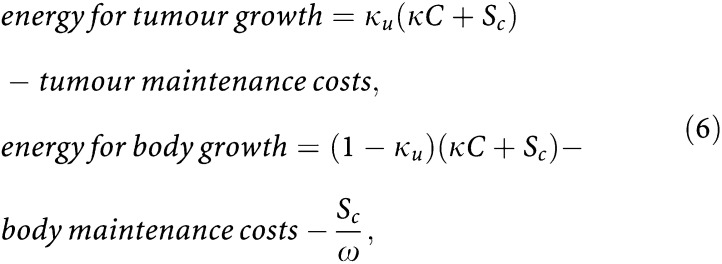


where *S*_*c*_ represents the energy obtained from the cachexia-related degradation of structural biomass. The coefficient *ω* is again the efficiency of energy regain. In the second equation, the term *S*_*c*_/*ω* stands for the actual costs of the shrinking process for the host. For simplicity, we assume that the cachectic degradation of host tissues occurs at a rate proportional to tumour size: *σ*_*u*_*V*_*u*_, where *σ*_*u*_ indicates the cachectic potency of a tumour (i.e., unit structure degraded per unit tumour volume per unit time). If *σ*_*u*_>0, the cachexia-mediated degradation of structural biomass results in an energy release rate of *S*_*c*_=*ω*[*G*]*σ*_*u*_*V*_*u*_. In contrast, if *σ*_*u*_=0 the tumour lacks any cachectic potency and the expressions above reduce to equations (3). Owing to the energy demand of the tumour and to the cachexia-mediated degradation of structural biomass, the host's energy balance will soon become negative. The host then has to degrade additional structural biomass to continue satisfying both the tumour's energy demand and its own maintenance requirements:


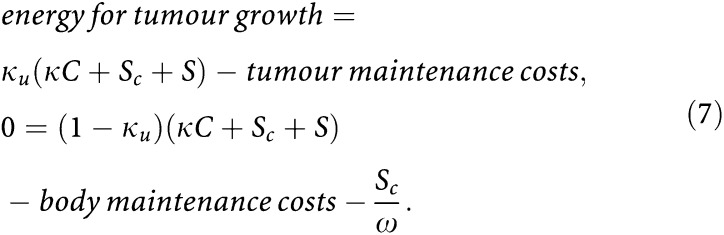


Figure 8

**Figure 8:**
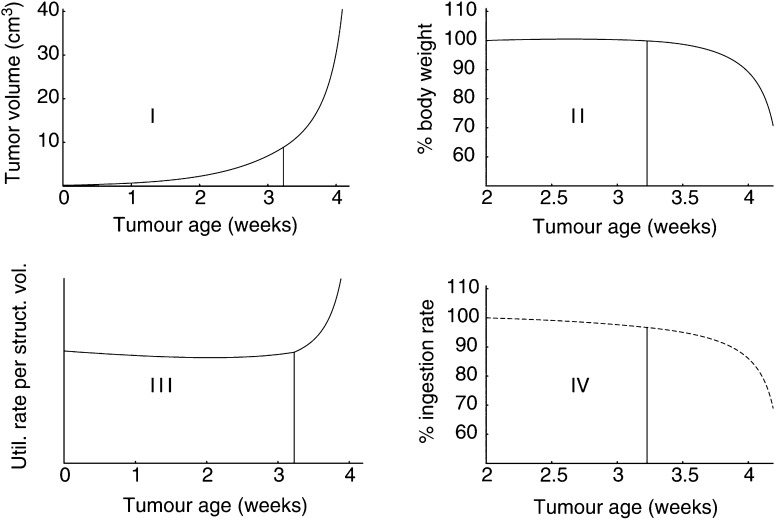
Implications of cachexia-mediated body-weight loss for tumour and host. (I) Tumour volume as a function of tumour age; (II) body weight as a fraction (in %) of body weight 2 days after tumour transplantation; (III) predicted energy expenditure per structural biomass; and (IV) food consumption as a fraction (in %) of the ingestion rate 2 days after tumour implantation. The vertical lines indicate the moment at which tumour-mediated loss of structural biomass starts. Tumour transplantation took place at age *t*_*i*_=15 weeks. Tumour parameters: [*M*_*u*_]<[*M*], [*G*_*u*_]<[*G*], *μ*_*u*_>1, and *σ*_*u*_>0.

shows the implications of cachexia for both host and tumour. The tumour type represented here has higher growth costs than the tumour type displayed in Figure 5. This explains the lower initial tumour growth rate in Figure 8-I. Nevertheless, the tumour is eventually more aggressive due to its capacity to cause cachexia. Indeed, the host starts to lose structural biomass 3.2 weeks after tumour transplantation. Moreover, a critical 30% body-weight loss is reached just 1 week later (see Figure 8-II). Figure 8-II also shows that, although we did not incorporate anorexia into the model, we predict a decrease in food consumption related to cachexia. Indeed, food intake diminishes progressively to match the lowered body weight. Figures 8-III and -IV reveal that an increased energy expenditure per structural biomass occurs despite the reduced food consumption. An elevated resting energy expenditure has been frequently observed in relation to cancer cachexia (Toomey *et al*, 1995; Emery, 1999; Bosaeus *et al*, 2002).

Above we argued that body-weight loss depends on the host physiological parameters (e.g., Figure 7). The same dependence holds for tumours with a cachectic potential. The time delay between tumorigenesis and disease onset, for example, may significantly vary among hosts. Consequently, the moment of disease onset nor the extent of the disease can be deduced from tumour size.

## DISCUSSION

The main difference between our approach and previous modelling approaches to tumour growth is that a tumour is conceived as a body part of the host rather than as an independent entity with an intrinsic maximum size. Our approach has the advantage that it can be used not only to describe tumour growth, but also to explore the relevance of interactions between tumour and host. We exemplified this by studying the influence of several host features on tumour behaviour and *vice versa*.

Another difference between our approach and others is that it does not assume *a priori* the existence of an asymptotic maximum tumour size. In contrast, for the widely applied Gompertz model (Winsor, 1932; Laird, 1964), maximum tumour size constitutes a model parameter and the associated S-shaped saturating growth pattern is an intrinsic property of the tumour. But not all tumours show saturating growth. The absence of a plateau in certain tumour growth data has been attributed to the early death of the host (Friberg and Mattson, 1997). That is, the host dies before tumour growth saturates. We doubt whether this is a solid explanation for any fast-growing tumour that does not deviate from an exponential growth pattern. But whether or not our doubt is justified, there is good reason not to assume *a priori* the existence of a maximum tumour size. Such an assumption hinders the possibility to predict under what physiological conditions a saturating tumour growth can be expected, and how the maximum tumour size depends on host and tumour characteristics.

We analysed the relation between shape of the tumour growth curve and the parameters of the host. Existence of a maximum tumour size is only expected for tumours whose maintenance costs and capacity to extract nutrients from blood satisfy the condition [*M*_*u*_]=*μ*_*u*_[*M*]. As this condition concerns tumour and host parameters, the shape of the tumour growth curve is determined by the energetic characteristics of both tumour and host.

Various factors known to affect tumour growth are not accounted for by our model, for example, diffusion-limited nutrient availability, immune response or the presence of growth inhibitors. The main reason for this is that when multiple determinants of tumour growth are incorporated at once, it is very difficult to pinpoint the impact of any determinant in particular. Our approach allowed us, for instance, to show that tumour–host interactions in energy dynamics may already cause tumour growth to saturate. This implies that diffusion-limited nutrient availability may be sufficient (e.g., Afenya and Calderón, 2000), but not essential to explain an S-shaped growth pattern. If we had included reaction-diffusion of nutrients from the outset, it would have been well-nigh impossible to arrive at this conclusion. To describe accurately the growth of particular tumours, however, it may be important to take specific features into account. An advantage of our model is that it can easily be extended to do so. In Appendix C, we exemplify this by showing how our model can be used to describe the growth of solid tumour with a necrotic kernel.

There is general agreement about the main causes of age-dependency of cancer incidence. However, this does not hold for the mechanisms underlying age-dependent tumour progression. Among the mechanisms proposed to explain the latter phenomenon are changes in angiogenic capacity (Pili *et al*, 1994), altered apoptotic cell death (Itzhaki *et al*, 2000), and immune senescence (Prehn, 1972; Tsuda *et al*, 1987). As results from various experiments provide evidence for different hypotheses, we preliminarily conclude that several aspects of the natural ageing process may affect tumour progression. On the basis of our model predictions, we hypothesise that the age-dependent energetic state of the host also plays an important role in determining tumour behaviour. Indeed, we argued that age-related differences in tumour growth are due to an age-associated decrease in energy expenditure per structural biomass.

We carried out a theoretical caloric restriction study to investigate the dependence of food consumption on a tumour's growth capacity. Model simulations suggested a strong dependence if tumorigenesis occurs after short-term caloric restriction. In contrast, a weak dependence of tumour growth on caloric intake is expected if tumorigenesis takes place after long-term exposure to caloric restriction. The dependence of tumour growth on food consumption can be understood on the basis of changes in the host energy expenditure.

With regard to the influence of a tumour on host physiology, we focused on tumour-mediated body-weight loss. Computer simulations revealed that body-weight loss cannot be unequivocally linked to the increase in tumour size. The main reason is that the severity of body-weight loss is determined by the energetics of both host and tumour, rather than by the tumour alone. Moreover, part of the energy released is actually wasted. These model outcomes may well explain the observation by Plata-Salamán (2000) that body-weight loss is often more dramatic than one would expect on the basis of the measured tumour growth.

To illustrate the clinical utility of our model, we applied it to understand the energetics behind cancer cachexia. From an energetic point of view, cachexia involves several metabolic alterations, among which are an increase in energy expenditure, a decrease in both structural biomass and reserves, and a reduced food consumption. As a result, the host is maintained in a negative energy balance. In the context of our modelling approach, diminished food consumption is a consequence rather than a cause of body-weight loss in cachexia. Yet, in response to the decreased food intake, an acceleration of body-weight loss occurs. From the obtained model predictions, we conclude that the extent of the disease as well as the time delay between tumorigenesis and disease onset strongly depend on the physiological features of the host.

A promising line of research would be to extend the model to include clinical interventions intended to reverse body-weight loss in tumour-bearing patients, such as food intake manipulations and parenteral nutritional support. Food intake manipulations can be incorporated, for instance, as an increase in the assimilation rate. Popp *et al* (1983) said that ‘the goal of nutritional therapy in the tumour-bearing host is support of the host carcass in the absence of increased tumour growth.’ Different food intake manipulations can be analysed with aid of our model, to figure out which manipulation may achieve that goal.

Several authors discussed the possible benefits of a low-fat dietary intervention in cancer patients (Rose *et al*, 1991; Mukherjee *et al*, 2002). As both tumour and host may grow slower or even shrink as a response to the decreased caloric intake, the main issue is whether the tumour or the host suffers more from the effects of caloric restriction. As our model accounts for food consumption, it can be used to examine the implications of such a dietary intervention.

Lazo (1985) argued that ‘the tumour cell population has to be viewed within the cell community that constitutes the organism.’ In line with this insight, we formulated our mathematical model within a framework describing the host. We applied the new model to explore several interactions between host and tumour, and were able to capture a number of empirically observed events. Moreover, for some of them we were able to provide an explanation based on energetic features of both tumour and host.

## Appendix A

### MODEL EQUATIONS

#### Tumour-free individual

We assume that the assimilation efficiency is independent of the food ingestion rate (Kooijman, 2000). If an animal receives a fixed fraction ϱ of *ad libitum* food consumption, its assimilation rate is then given by: *A*=ϱ*A*_*m*_, where *A*_*m*_ denotes the maximum (diet-composition specific) assimilation rate and ϱ is the so-called food-supply coefficient. We define the surface-specific maximum assimilation rate as: {*A*_*m*_}=*A*_*m*_*V*_1∞_^−2/3^, with *V*_1∞_ being the *ad libitum* asymptotic maximum structural volume. The assimilation rate can thus be written as: *A*=ϱ{*A*_*m*_}*V*_1∞_^2/3^

According to the DEB theory, the utilisation rate is given by

where *E* denotes the amount of reserves and *v*={*A*_*m*_}/[*E*_*m*_] is the energy conductance, with [*E*_*m*_] being the maximum reserve density. The change in the amount of reserves is then given by the difference between assimilation and utilisation (see Figure 1), that is: d*E*/d*t*=*A*−*C*. Substitution of the expressions for *C* into this equation leads to

with *e* being the scaled energy density, *e*=*E*/[*E*_*m*_]*V*. At the beginning of the study, the host's age is *t*_0_ weeks and its initial reserve density is *e*(*t*_0_)=*e*_0_.

In the context of the DEB model (Figure 1), the body has two components. Total body weight is therefore a function of both structure and reserves: *W*=*d*_*V*_(1+*ξe*)*V*, where *d*_*V*_ is the density of structural biomass and *ξ* is a dimensionless compound parameter (Van Leeuwen *et al*, 2002). As explained in the body of the article, the change in structural volume is given by equation (2), which can be written as

where *g*=[*G*]/*κ*[*E*_*m*_] is the energy-investment ratio and *m*=[*M*]/[*G*] is the the maintenance-rate coefficient. Substitution of the expression for *C* (equation (A.1)) gives

From equations (A.2) and (A.4), it can be shown that *V*(*t*) tends to an asymptotic maximum value, *V*_ϱ∞_, which satisfies *V*_ϱ∞_=ϱ*V*_1∞_=ϱ(*v*/ϱ*m*)^3^=ϱ(*κ*{*A*_*m*_}/[*M*])^3^. Consequently, *A*=ϱ^1/3^{*A*_*m*_}*V*_ϱ∞_^2/3^ and the scaled reserve density can be expressed as

In sum, the change in size of a tumour-free organism is characterised by equations (A.4) and (A.5), with initial conditions *V*(*t*_0_)=*V*_0_ and *e*(*t*_0_)=*e*_0_.

#### Tumour-bearing individual

If tumorigenesis (or tumour implantation) happens at time *t*_*i*_, let *V*_*ui*_ denote the initial tumour size. At *t*_*i*_, the host's structural body volume is *V*_*i*_=*V*(*t*_*i*_) and its reserve density *e*(*t*_*i*_)=*e*_*i*_. As the tumour appropriates reserves originally destined to be spent on physiological processes such as body growth, the host is no longer able to reach its maximum size. To account for this, we generalised the expressions for the scaled reserve density (equation (A.5)) and the assimilation rate:

where  is defined as the ‘expected’ ultimate structural biomass predicted at time *t*. We assume that 𝒱_ϱ∞_ is a function of tumour volume:

For a tumour-free animal in the diet group ϱ, the function

is constant and equal to

.

For both tumour and host, we assume that growth costs are proportional to the increase in structural volume, whereas the maintenance costs are proportional to structural volume. Consequently, the generalised *κ*-rule (equations (3)) can be written as

where *g*_*u*_=[*G*_*u*_]/*κ*[*E*_*m*_] and *m*_*u*_=[*M*_*u*_]/[*G*_*u*_]. The expression for *κ*_*u*_ is given in equation (4). In the absence of a tumour, equation (A.8) reduces to equation (A.3). Substitution of the expression for the utilisation rate (equation (A.1)) into the equations above leads to

These equations, together with equation (A.6) and the initial conditions *V*(*t*_*i*_)=*V*_*i*_, *V*_*u*_(*t*_*i*_)=*V*_*ui*_, and *e*(*t*_*i*_)=*e*_*i*_, specify the change in size of both host and tumour. If the condition *m*_*u*_*g*_*u*_=*μ*_*u*_*mg* holds, the tumour grows according to an S-shaped pattern. Moreover, this condition marks the bifurcation between tumours growing (*m*_*u*_*g*_*u*_<*μ*_*u*_*mg*) or dying off (*m*_*u*_*g*_*u*_>*μ*_*u*_*mg*).

As explained in the body of the article, equations (A.10) and (A.11) are reliable thermodynamically as long as d*V*/d*t*⩾0. Let *t*_*s*_ denote the time (age) at which increase in structure ceases. We define *V*_*s*_=*V*(*t*_*s*_), *V*_*us*_=*V*_*u*_(*t*_*s*_), and *e*_*s*_=*e*(*t*_*s*_). For *t*⩾*t*_*s*_ the following equations, together with equation (A.6), describe the loss of structural body mass and the increase in tumour size:

with initial conditions *V*(*t*_*s*_)=*V*_*s*_, *V*_*u*_(*t*_*s*_)=*V*_*us*_ and *e*(*t*_*s*_)=*e*_*s*_. Equations (A.12) and (A.13) result from the substitution of the expression for *C* (equation (A.1)) and *S*=−*ω*[*G*]d*V*/d*t* into equations (5). Notice that if the condition *m*_*u*_*g*_*u*_=*μ*_*u*_*mg* holds, we have d*V*_*u*_/d*t*=0.

#### Cachexia equations

Substitution of the expression for *C* (equation (A.1)) and *S*_*c*_=*ω*[*G*]*σ*_*u*_*V*_*u*_, into equations (6) gives:

These equations, together with equation (A.6) and initial conditions *V*(*t*_*i*_)=*V*_*i*_, *V*_*u*_(*t*_*i*_)=*V*_*ui*_, and *e*(*t*_*i*_)=*e*_*i*_, specify the change in the body size and in tumour volume. If *σ*_*u*_=0, equations (A.14) and (A.15) reduce to equations (A.10) and (A.11), respectively. Let *t*_*s*_ denote the time (age) at which equation (A.14) satisfies (d*V*/d*t*)*t*_*s*_=0. For *t*⩾*t*_*s*_, the following equations apply:

The initial conditions at *t*_*s*_ are determined by equations (A.6), (A.14) and (A.15). Equations (A.16) and (A.17) result from the substitution of the expression for *C* (equation (A.1), *S*=*ω*[*G*]d*V*/d*t* and *S*_*c*_=*ω*[*G*]*σ*_*u*_*V*_*u*_ into equations (7). If *σ*_*u*_=0, equations (A.16) and (A.17) reduce to equations (A.12) and (A.13), respectively.

## Appendix B

### PARAMETER VALUES

Hubert *et al* (2000) consider three different feeding regimes, *ad libitum* (ϱ=1), 25% caloric restriction (ϱ=0.75), and 55% caloric restriction (ϱ=0.45). The animals were 35 days (5 weeks) old at study initiation. As the rats were split into three groups at the beginning of the study, the values of *W*_0_=*W*(*t*_0_) and *e*_0_=*e*(*t*_0_) can be assumed to be the same for any diet group. Moreover, because all animals received *ad libitum* feeding until the beginning of the caloric-restriction study, the assumption *e*_0_=1 holds. In addition, we fixed *d*_*V*_ on a value of 1 g cm^−3^. During the least-square fitting procedure, we only varied the value of the food-supply coefficient among the different diet groups. The estimated parameter values are: *ḡ*=7.1, W*¯*_0_=142.84 g, ξ=0.94, *V̄*_1∞_=436.93 cm^3^ and *v̄*=2.22 cm^−1^week. Consequently: *m̄*=*v̄*/*ḡ* (*V̄*_1∞_)^−1/3^=0.041 week^−1^. The body growth curves corresponding to the estimated parameter values are shown in Figure 2.

For any displayed tumour: *V*_*ui*_=0.2 cm^3^. All computer simulations involved a ‘switch’ of equations at time *t*_*s*_, with *t*_*s*_ the time (age) at which loss of structural biomass begins, that is, (d*V*/d*t*)*t*_*s*_=0.

Figure 4 (shape of the tumour growth curve): For any tumour: *ω*=0.75 and *σ*_*u*_=0 week^−1^.

Solid line: *μ*_*u*_=4, *g*_*u*_=3.5, and *m*_*u*_=*μ*_*u*_*mg*/*g*_*u*_≈0.33 week^−1^.

Broken line: *μ*_*u*_=2, *g*_*u*_=2.1, and *m*_*u*_=0.14 week^−1^.

Dotted line: *μ*_*u*_=1, *g*_*u*_=2.1, and *m*_*u*_=0.027 week^−1^.

Figure 5 (influence of host age on tumour growth): *μ*_*u*_=9, *g*_*u*_=5.1, *m*_*u*_=10^−3^ week^−1^, *ω*=0.5, and *σ*_*u*_=0 week^−1^.

Figure 6 (effect of caloric restriction on tumour growth): *μ*_*u*_=3, *g*_*u*_=*g*, *m*_*u*_=*m*, *ω*=0.75, and *σ*_*u*_=0 week^−1^.

Figure 7 (tumour-mediated body-weight loss): *μ*_*u*_=9, *g*_*u*_=5.1, *m*_*u*_=10^−3^ week^−1^, *ω*=0.5, and *σ*_*u*_=0 week^−1^ (same values as in Figure 5).

Figure 8 (implications of cachexia-mediated body-weight loss for tumour and host): *μ*_*u*_=9, *g*_*u*_=6.1, *m*_*u*_=0.01 week^−1^, *ω*=0.5, and *σ*_*u*_=1 week^−1^.

## Appendix C

### MODEL EXTENSION

An important advantage of our modelling approach is that it can be easily extended to account for specific features of a particular tumour. To exemplify this, we show how it can be used to describe the growth of a tumour with a dead kernel. For simplicity, we assume that the the whole tumour is spherical in shape. When the tumour reaches a critical size, defined by a radius *δ*_*m*_, the tumour starts to develop a dead kernel. In mathematical terms, this implies that an additional cause of tumour-cell death has to be added to our model.

Let us denote as

our expression for the change in tumour volume (equations (A.11), (A.13), (A.15), or (A.17)). The growth of the viable cell population in the tumour developing a dead kernel is then as follows:

where

represents the death of tumour cells due to insufficient nutrient availability within the tumour. We assume that the volume of cells starved to death give rise to an equal volume of dead biomass. As the necrotic core can only increase by death of cells in the living layer (Mayneord, 1932), we then have that

,with *V*_†_ the volume of dead biomass. Substitution of this expression for

in equation (C.1), leads to

As the total volume of the tumour satisfies *V*_*T*_=*V*_*u*_+*V*_†_, the expression above is equivalent to

).

As the whole tumour is spherical in shape:

,

with *L*_*T*_ being the radius of the tumour. From derivating this expression, we obtain

To describe exhaustively the growth of the whole tumour, we now have to fill in the expression for *V*_*u*_ in the equation above. If we assume that the thickness of the living layer remains constant during tumour growth, the radius of the dead kernel is given by *L*_†_=*L*_*T*_−*δ*_*m*_, and

because *V*_*u*_=*V*_*T*_−*V*_†_ and

.

Equation (C.3) together with equation (C.2) describes the change in the radius of a tumour with a necrotic core. In the particular case that the living biomass grows exponentially (i.e.,

,

these equations reduce to the tumour growth equation proposed by Mayneord (1932).

## References

[bib1] Adam JA, Bellomo N (eds) (1997) A Survey of Models for Tumor-immune System Dynamics. Boston: Birkhäuser

[bib2] Afenya EK, Calderón CP (2000) Diverse ideas on the growth kinetics of disseminated cancer cells. Bull Math Biol 62: 527–542 10812720 10.1006/bulm.1999.0165

[bib3] Von Bertalanffy L (1957) Quantitative laws in metabolism and growth. Q Rev Biol 32: 217–231 13485376 10.1086/401873

[bib4] Bosaeus I, Daneryd P, Lundholm K (2002) Dietary intake, resting energy expenditure, weight loss and survival in cancer patients. J Nutr 132: 3465–3466 10.1093/jn/132.11.3465S 12421871

[bib5] Cameron IL, Pavlat WA, Stevens MD, Rogers W (1979) Tumor–host responses to various nutritional feeding procedures in rats. J Nutr 109: 671–684 107299 10.1093/jn/109.4.671

[bib6] Canolty NL, Koong LJ (1976) Utilization of energy for maintenance and for fat and lean gains by mice selected for rapid postweaning growth rate. J Nutr 106: 1202–1208 940001 10.1093/jn/106.8.1202

[bib7] Dewanji A, Moolgavkar SH, Luebeck EG (1991) Two-mutation model for carcinogenesis: joint analysis of premalignant and malignant lesions. Math Biosci 104: 97–109 1804458 10.1016/0025-5564(91)90032-e

[bib42] Donin N, Sinai J, Staroselsky A, Mahlin T, Nordenberg J, Leibovici J (1997) Comparison of growth rate of two B16 melanomas differing in metastasic potential in young versus middle-aged mice. Cancer Investigations 15: 416–421 10.3109/07357909709047580 9316623

[bib8] Duclaux, E (1898) Traité de microbiologie, chapter Vie a érobie et ana érobie 208–212. Paris: Masson et cie

[bib9] Emery PW (1999) Cachexia in experimental models. Nutrition 15: 600–603 10422096 10.1016/s0899-9007(99)00095-7

[bib10] Friberg S, Mattson S (1997) On the growth rates of human malignant tumors: implications for medical decision making. J Surg Oncol 65: 284–297 9274795 10.1002/(sici)1096-9098(199708)65:4<284::aid-jso11>3.0.co;2-2

[bib11] Groebe K, Mueller-Klieser W (1991) Distributions of oxygen, nutrient, and metabolic waste concentrations in multicellular spheroids and their dependence on spheroid parameters. Eur Biophys J 19: 169–181 2029873 10.1007/BF00196343

[bib12] Holmes F (1989) Clinical evidence for a change in tumor aggressiveness with age. Semin Oncol 16: 34–40 2645648

[bib13] Hubert MF, Laroque P, Gillet JP, Keenan KP (2000) The effects of diet, *ad libitum* feeding, and moderate and severe dietary restriction on body weight, survival, clinical pathology parameters, and cause of death in control Sprague–Dawley rats. Toxicol Sci 58: 195–207 11053556 10.1093/toxsci/58.1.195

[bib14] Itzhaki O, Skutelsky E, Kaptzan T, Siegal A, Michowitz, Sinai J, Huszar M, Nafar S, Leibovici J (2000) Macrophage-recognized molecules of apoptotic cells are expressed at higher levels in AKR lymphoma of aged as compared to young mice. In book: The Biology and Pathology of Innate Immunity Mechanism, Keisari Y, Ofek I (eds). In Series: Volume 479 of Advances in Experimental Medicine and Biology Vol. 47. Dordrecht: Kluwer Academic Publishers 10.1007/0-306-46831-X_22 10897426

[bib15] Kooijman SALM (1988) The Von Bertalanffy growth rate as a function of physiological parameters; a comparative analysis. In Mathematical Ecology, Hallam TG, Gross LJ, Levin SA (eds). Singapore: World Scientific

[bib16] Kooijman SALM (2000) Dynamic Energy and Mass Budgets in biological systems. Theory and applications. Cambridge: Cambridge University Press

[bib17] Kooijman SALM (2001) Quantitative aspects of metabolic organization; a discussion of concepts. Philos Trans R Soc London 356: 331–349 11316483 10.1098/rstb.2000.0771 PMC1088431

[bib18] Laird, AK (1964) Dynamics of tumor growth. Br J Cancer 18: 490–502 10.1038/bjc.1964.55 PMC2071101 14219541

[bib19] Lazo PA (1985) Tumour–host metabolic interaction and cachexia. FEES Lett 187: 189–192 10.1016/0014-5793(85)81239-4 4018258

[bib20] Van Leeuwen IMM, Kelpin FDL, Kooijman SALM (2002) A mathematical model that accounts for the effects of caloric restriction on body weight and longevity. Biogerontol 3: 373–381 10.1023/a:1021336321551 12510176

[bib21] Matthys P, Billiau A (1997) Cytokines and cachexia. Nutrition 13: 763–770 9290087 10.1016/s0899-9007(97)00185-8

[bib22] Mayneord MV (1932) On a law of growth of Jensen's rat sarcoma. Am J Cancer 16: 841–846

[bib23] Mukherjee P, El-Abbadi MM, Kasperzyk JL, Ranes MK, Seyfriend TN (2002) Dietary restriction reduces angiogenesis and growth in an orthotopic mouse brain tumour model. Br J Cancer 86: 1615–1621 12085212 10.1038/sj.bjc.6600298 PMC2746602

[bib24] Payne PR, Waterlow JC (1971) Relative energy requirements for maintenance, growth, and physical activity. Lancet 2: 210–211 4104867 10.1016/s0140-6736(71)90917-2

[bib25] Peer PG, Dijck JA, Hendriks JH, Verbeek AL (1993) Age-dependent growth rate of primary breast cancer. Cancer 71: 3547–3551 8490903 10.1002/1097-0142(19930601)71:11<3547::aid-cncr2820711114>3.0.co;2-c

[bib26] Pili R, Guo Y, Chang J, Nakanishi H, Martin GR, Passaniti A (1994) Altered angiogenesis underlying age-dependent changes in tumor growth. J Natl Cancer Inst 86: 1303–1304 7520508 10.1093/jnci/86.17.1303

[bib27] Pirt SJ (1965) The maintenance energy of bacteria in growing cultures. Proc R Soc London 163: 224–231 4378482 10.1098/rspb.1965.0069

[bib28] Plata-Salamán CR (2000) Central nervous system mechanisms contributing to the cachexia-anorexia syndrome. Nutrition 16: 1009–1012 11054608 10.1016/s0899-9007(00)00413-5

[bib29] Popp MB, Wagner SC, Brito OJ (1983) Host and tumor responses to increasing levels of intravenous nutritional support. Surgery 94: 301–308 6410526

[bib30] Prehn RT (1972) The immune reaction as a stimulator of tumor growth. Science 176: 170–171 5014438 10.1126/science.176.4031.170

[bib31] Ramsey JJ, Harper ME, Weindruch R (2000) Restriction of energy intake, energy expenditure and aging. Free Radic Biol Med 29: 946–968 11084284 10.1016/s0891-5849(00)00417-2

[bib32] Rose DP, Connolly JM, Meschter CL (1991) Effect of dietary fat on human breast cancer growth and lung metastasis in nude mice. J Natl Cancer Inst 83: 1491–1495 1920496 10.1093/jnci/83.20.1491

[bib33] Sherman CD, Portier CJ (2000) Calculation of the cumulative distribution function of the time to a small observable tumor. Bull Math Biol 62: 229–240 10824428 10.1006/bulm.1999.0148

[bib34] Thomlison RH, Gray LH (1955) The histological structure of some human lung cancers and the possible implications for radiotherapy. Br J Cancer 9: 539 549 13304213 10.1038/bjc.1955.55 PMC2073776

[bib35] Tisdale MJ (2000) Metabolic abnormalities in cachexia and anorexia. Nutrition 16: 1013–1014 11054609 10.1016/s0899-9007(00)00409-3

[bib36] Tisdale MJ (2001) Cancer anorexia and cachexia. Nutrition 17: 438–442 11377146 10.1016/s0899-9007(01)00506-8

[bib37] Toomey D, Redmond HP, Bouchierhayes D (1995) Mechanisms mediating cancer cachexia. Cancer 76: 2418–2426 8625066 10.1002/1097-0142(19951215)76:12<2418::aid-cncr2820761204>3.0.co;2-c

[bib38] Tsuda T, Kim YT, Siskind, GW (1987) Role of the thymus and T-cells in slow growth of B16 melanoma in old mice. Cancer Res 47: 3097–3102 3495326

[bib39] Ward JP, King JR (1997) Mathematical modelling of avascular-tumour growth. IMA J Math Appl Med Biol 14: 39–69 9080687

[bib40] Ward JP, King JR (1999) Mathematical modelling of the effects of mitotic inhibitors on avascular-tumour growth. J Theor Med 1: 287–311

[bib41] Winsor CP (1932) The Gompertz curve as a growth curve. Proc Natl Acad Sci USA 18: 1–8 16577417 10.1073/pnas.18.1.1 PMC1076153

